# IL-17 and IL-23 in lupus nephritis - association to histopathology and response to treatment

**DOI:** 10.1186/s12865-015-0070-7

**Published:** 2015-02-12

**Authors:** Agneta Zickert, Petra Amoudruz, Yvonne Sundström, Johan Rönnelid, Vivianne Malmström, Iva Gunnarsson

**Affiliations:** Department of Medicine, Unit of Rheumatology, Karolinska University Hospital, Karolinska Institute, SE-171 76, Stockholm, Sweden; Department of Immunology, Genetics and Pathology, Uppsala University, Uppsala, Sweden

## Abstract

**Background:**

Recent studies indicate a central role for the IL-23/IL-17 axis in the pathogenesis of lupus nephritis (LN) but the importance in the context of treatment outcome is unknown. We studied various cytokines, including the IL-23/IL-17 axis, in association to histopathology and response to therapy.

**Methods:**

Fifty-two patients with active LN were included. Renal biopsies were performed at baseline and after immunosuppressive treatment. Serum levels of TNF-α, IFN-γ, IL-6, IL-10, IL-17, IL-23 and TGF-β were analysed at both biopsy occasions and in 13 healthy controls. IL-17 expression in renal tissue was assessed by immunohistochemistry. Biopsies were evaluated regarding WHO-classification and renal disease activity was estimated using the BILAG-index. Improvement of 2 grades in renal BILAG was regarded complete response, and 1 grade partial response.

**Results:**

At baseline, all patients had high disease activity (BILAG A/B). Baseline levels of IL-6, IL-10, IL-17, IL-23 (p < 0.001) and IFN-γ (p = 0.03) were increased in patients vs. controls. In contrast, TGF-β was lower in patients compared to controls (p < 0.001).

Baseline levels of IL-17 were higher in patients with persisting active nephritis (WHO III, IV, V) after treatment, i.e. a poor histological response, vs. WHO I-II (p < 0.03). At follow-up, IL-23 were higher in BILAG-non-responders vs. responders (p < 0.05). Immunostaining of renal tissue revealed IL-17 expression in inflammatory infiltrates.

**Conclusions:**

High baseline IL-17 predicted an unfavourable histopathological response, and BILAG-non-responders had high IL-23, indicating that that a subset of LN-patients has a Th-17 phenotype that may influence response to treatment and could be evaluated as a biomarker for poor therapeutic response.

## Background

Lupus nephritis (LN) is a severe manifestation of systemic lupus erythematosus (SLE), affecting up to 60% of SLE patients at some point of the disease [1]. The pathogenesis for LN is complex and involves multiple components of both the innate and adaptive immune systems. Although the exact mechanisms remain unclear, the hallmark in the pathogenesis is production of autoantibodies and immune complex formation with subsequent infiltration by inflammatory cells in renal tissue [2-4]. Many cytokines are also involved and contribute to both onset and progression of renal pathology.

Interferon (IFN)-α is regarded an important cytokine in SLE pathogenesis but several additional cytokines have been put forward in this context, including interleukin (IL)-6, IL-1, tumour necrosis factor (TNF)-α, IFN-γ, IL-12, IL-10, transforming growth factor (TGF)-β, IL-2 and more recently the IL-23/IL-17 axis [5-7].

Current studies indicate a central role for IL-17 in the pathogenesis of LN [8,9]. IL-17 is the main cytokine from Th17 cells, a T-cell subset evolved from CD4+ T-cells under the influence of IL-6, IL-21 and IL-1. It is also produced by other cells, such as T-cell receptor (TCR)γδ and TCRαβ double negative (DN) T-cells (CD3 + CD4-CD8-) [9]. IL-17 has strong proinflammatory effects [10], induces other cytokines, promotes recruitment of inflammatory cells and facilitates T-cell infiltration [9]. A synergy between IL-17 and B-cell activating factor (BAFF) in promoting B-cell proliferation and antibody production has also been shown [11]. Increased number of Th17 cells as well as high serum levels of IL-17 has been demonstrated in lupus patients [12-14] and IL-17 has been detected in glomeruli and in interstitial infiltrating T-cells [15-17].

IL-23 is released from antigen presenting cells and induces expansion of Th17 cells and is necessary for their maintenance [18], thus forming the IL-23/IL-17 axis. Previous studies found high serum levels of IL-23 in LN [13,17] and an expansion of T-cells expressing both high IL-17 and IL-23-receptor have been demonstrated in lupus-prone mice [8]. IL-23–treated lymphocytes have been shown to induce nephritis in mice [8] while IL-23-receptor deficiency has been found to prevent development of nephritis in murine lupus models [19].

Although evidence of the role for Th17 is accumulating, this has not been put in the context of response to immunosuppressive treatment in LN patients. Increased knowledge of cytokines in LN may contribute to further understanding of the pathogenesis and may lead to identification of new biomarkers for disease activity and to the development of new treatment strategies. Here we studied serum levels of several cytokines, including the role of Th17 cytokines, in both active disease and after immunosuppressive therapy in association to histopathological findings and response to therapy. We also performed tissue stainings for IL-17 in kidney biopsies from six LN patients.

## Methods

### Patients

Fifty-two patients with SLE and biopsy-proven active LN between1996 and 2009 were included. All patients fulfilled the updated revised American College of Rheumatology (ACR) classification criteria for SLE [20]. As part of the clinical routine at our unit, all patients underwent a second renal biopsy following immunosuppressive induction therapy after a median time of 7 months (range 5–15). Clinical data, blood and urinary samples were collected at both biopsy occasions. Serum samples were stored at −70°C. Thirteen healthy blood donors, 11 females and 2 males mean age 36 years (range 18–60), were used as control group for the cytokine analyses. Written informed consent was obtained from all subjects and the study protocol was approved by the regional ethics committee in Stockholm (KI Forskningsetikkommitte Nord, Karolinska sjukhuset; Regionala etikprövningsnämnden i Stockholm).

### Evaluation of renal function, histopathology and renal activity

Renal evaluation at the time of both biopsies included urine analyses (dipslide procedure) and investigation of 24-hour urine albumin excretion. Renal function was determined by serum creatinine levels (μmol/l).

Renal biopsies were evaluated by light microscopy, immunofluorescence and electron microscopy and were graded according to the World Health Organization (WHO) classification of nephritis [21] and scored for activity and chronicity indices [22]. WHO class I or II at follow-up was regarded as good histopathological response whereas WHO III-IV and V as non-response.

As control, renal tissue from an unaffected part of a kidney nephrectomised due to renal carcinoma was used.

Evaluation of renal disease activity was estimated using the British Isles Lupus Assessment Group (BILAG) index [23]. Renal disease activity according to BILAG is defined as A, B, C or D according to levels of proteinuria, creatinine and blood pressure and also based upon findings on urinary sediment and histological data (as defined in [23]). An improvement of at least two grades in the renal domain of BILAG (i.e. from A to C or B to D) at follow-up was regarded as complete response (CR), whereas an improvement of one grade was regarded as partial response (PR) and no improvement as non-response (NR).

### Serology and complement measures

Assessments of serum IgG anti-dsDNA antibodies were carried out by immunofluorescence microscopy using *Crithidiae luciliae* as source of antigen. Analyses of complement component C3 (normal range 0.67-1.43 g/l) and C4 (normal range 0.12-0.32 g/l) levels were determined by nephelometry.

### Cytokine analyses

All serum samples for cytokine measurements were kept at −70°C until use. TNF-α, IFN-γ, IL-6 and IL-10 were measured using the Cytometric Bead Array (CBA), (BD Biosciences Pharmingen, USA) technique. Briefly, the standards and samples were mixed with phycoerythrin-labeled beads and incubated for 3 h. After incubation, the samples and standards were washed to remove unbound material and analyzed using the BD/CBA software. After discussions with the manufacturer we extended the standard curve by diluting the standard further than indicated in the protocol. Hereby, the detection limits of our assay were: IL-6 (1.0 pg/ml), IL-10 (1.5 pg/ml), IFN-γ (4.1 pg/ml) and TNF-α (1.5 pg/ml).

IL-17, IL-23 and TGF-β were measured with ELISA (R&D Systems, Abingdon, UK). We extended the standard curve for IL-17 and IL-23 further than indicated in the protocol. The detection limits were 6.6 and 1.3 pg/ml respectively.

For statistical reasons, all undetectable levels were set to half of the detection level.

### Immunostaining of renal biopsies

Immunohistochemical stainings of IL-17 expression was performed on formaldehyde-fixed paraffin-embedded serial 4 μm sections of 6 renal biopsies from patients with LN and from control renal tissue. Slides were deparaffinised in xylene and rehydrated to water with ethanol. Antigen retrieval was achieved by microwave irradiation prior to staining. Briefly, tween20 was used to permeabilize the cells, endogenous peroxidase was blocked with H_2_O_2_, avidin-biotin blocking (DAKO, Glostrup Denmark) was used, and unspecific antibody staining was blocked using skim milk, Poly-L-Lysine (Sigma-Aldrich, St Louis, MO, USA) donkey and human serum. Antibodies used were mouse anti-IL17 (R&D Systems, Minneapolis, MN, USA), rabbit anti-CD3 (Thermo Scientific, Rockford, IL, USA) and isotype controls, mouse IgG1 (R&D Systems, Minneapolis, MN, USA) and rabbit (DA1E) mAb IgG XP® Isotype Control (Cell Signaling Technology, Inc., Boston, MA, USA). Biotin-labelled donkey anti-rabbit- and donkey anti-mouse secondary antibody (Jackson ImmunoResearch Laboratories, Inc., West Grove, PA, USA) were used for detection. For signal amplification Peroxidase conjugated Avidin Biotin Complex (Vector Laboratories Inc., Burlingame, CA, USA) was used in combination with Biotin conjugated Tyramide (TSA indirect blot), (PerkinElmer, Inc., Waltham, MA, USA). Stainings were developed using DAB (Sigma-Aldrich) dissolved in trisbuffer saline. Sections were counterstained with Mayer’s hematoxylin.

### Statistics

For comparisons of variables at baseline and follow-up the nonparametric Wilcoxon matched pair test was used. For comparisons of variables between two groups the non-parametric Mann–Whitney test was used and for comparisons between multiple groups the Kruskal-Wallis test was used. Correlations were calculated using Spearman’s rank correlation. Statistical significance was set at the level of p < 0.05. Statistical evaluation was made by statistical software, STATISTICA 9, StatSoft, Tulsa, USA.

## Results

### Renal activity and histopathology

All patients had an active nephritis at baseline with biopsies showing WHO class III (n = 15), III/V (n = 4), IV (n = 24), V (n = 8) and one glomerular vasculitis. All patients had high renal disease activity, 49/52 had renal BILAG A and 3/52 BILAG B. Forty-six of the patients were female (88%) and 6 were men (12%), mean age was 32 years (range 18–59).

The patients were treated in accordance with standard therapy for LN; corticosteroids combined with intravenous cyclophosphamide (n = 40), mycophenolate mofetil (n = 9) or rituximab (n = 2). One patient was treated with azathioprin. The treatment regimen for cyclophosphamide was 0.5-1 g/m^2^ monthly as modified from the NIH-protocol [24]. At the time for the first renal biopsy, 36/52 (69%) of the patients were treated with prednisolone, median dose 20 mg/day (range 2.5-60). At start of immunosuppressive therapy, all but one (98%) were treated with prednisolone with median dose 40 mg/day, doses ranging from 2.5 to 80 mg/day as decided by the treating physician, and were thereafter successively tapered. At repeat biopsies, 98% of the patients were still treated with prednisolone, median dose 10 mg/day (range 2.5-30).

Follow-up biopsies showed WHO class I (n = 1), II (n = 18), III (n = 9), III/V (n = 1), IV (n = 8) and V (n = 14) and one had developed a renal vasculitis (seen in a patient with class III at first biopsy). The patient with a vasculitis pattern at first biopsy had class II at repeat biopsy. Nineteen patients (36%) were regarded histopathological responders (class I/II). The renal activity index decreased significantly (<0.001) whereas there was an increase in the chronicity index (p < 0.001).

Levels of C3 and C4 increased (p < 0.001) and there was a decrease in proteinuria at follow-up (p < 0.001) whereas no overall difference in creatinine levels was found.

At follow-up, 8/52 had renal BILAG A, 21/52 BILAG B, 11/52 BILAG C and 12/52 had BILAG D. Twenty-two patients (42.3%) were regarded as CR, 20/52 (38.5%) as PR and 10/52 (19.2%) were regarded NR according to BILAG. The clinical characteristics of patients and nephritis data at baseline and follow-up are presented in Table 1.

**Table 1:** **Patient characteristics at baseline and follow-up**

	**Baseline**	**Follow-up**	**p-value**
**Creatinine, μmol/l, median**	82 (52–284)	77 (48–306)	ns
**Proteinuria, g/d, median**	1.45 (0–8.4)	0.5 (0–3.6)	*<0.001*
**C3, g/l, median**	0.5 (0.12-1.13)	0.74 (0.36-1.41)	*<0.001*
**C4, g/l, median**	0.11 (0.02-0.51)	0.13 (0.02-0.45)	*0.002*
**Anti-DNA-ab positivity** ^#^ **, %**	84	57	
**Nephritis class, WHO (n)**			
**I-II**	-	19	
**III**	15	9	
**III/V**	4	1	
**IV**	24	8	
**V**	8	14	
**Vasculits**	1	1	
**Activity index, median**	5 (0–13)	2 (0–12)	*<0.001*
**Chronicity index, median**	1 (0–6)	1 (0–8)	*<0.001*
**Treatment (n)**			
**Cyclophosphamide**	40		
**Mycophenolate mofetil**	9		
**Rituximab**	2		
**Azathioprin**	1		
**Prednisolone dose, median**	40 (2.5-80)	10 (2.5-30)	
**BILAG** ^**†**^ **, renal**			
**A**	49	8	
**B**	3	20	
**C**		12	
**D**		12	

Range are given in parenthesis, ^#^Anti-dsDNA raised to at least 1:25, ^†^BILAG, British Isles Lupus Assessment Group.

### Serum cytokines

Most baseline levels of cytokines were increased in patients vs. controls, this was highly significant for IL-6, IL-10, IL-17, IL-23 (p < 0.001 for all) and also significant for IFN-γ (p = 0.03). TGF-β was lower in patients vs. controls (p < 0.001).

Overall cytokine levels decreased after treatment, this was significant for IL-6 (p < 0.001), IL-10 (p = 0.02) and IL-17 (p = 0.01), for IL-23 there was a trend towards lower levels at follow-up (p = 0.06). For TNF-α and IFN-γ there was an overall decrease in serum levels although not statistically significant (ns), while for TGF-β an increase was documented (p = 0.005). No difference in cytokine levels was found comparing the different immunosuppressive treatments or doses of prednisolone at first and repeated biopsies (data not shown). Cytokine levels in patients and controls are presented in Table 2.

**Table 2:** **Baseline levels of cytokines in patients at baseline vs. controls, and baseline vs. follow-up levels in patients**

	**Patients, baseline median (range)**	**Patients, follow-up median (range)**	**Controls median (range)**	***p***
**IL-6, pg/ml**	10.02 (0.62-81.78)		2.18 (0.62-4.64)	*<0.001*
		3.29 (0.62-30.72)		*<0.001*
**IL-10, pg/ml**	9.78 (0.54-81.04)		0.54 (0.54-3.90)	*<0.001*
		4.95 (0.54-37.21)		*<0.002*
**TNF-α, pg/ml**	1.24 (0.55-12.51)		0.55 (0.55-4.29)	0.10
		1.00 (0.55-78.22)		0.27
**IFN-γ, pg/ml**	7.05 (0.52-338.9)		0.52 (0.52-8.97)	*0.03*
		6.63 (0.52-278.2)		0.75
**IL-17, pg/ml**	97.42 (3.30-381.6)		3.30 (3.30-62.66)	*<0.001*
		47.98 (3.30-824.5)		*0.01*
**IL-23, pg/ml**	5.52 (0.66-121.5)		0.66 (0.66-0.66)	*<0.001*
		3.27 (0.66-32.67)		0.06
**TGF-β, ng/ml**	42.99 (10.22-85.94)		82.71 (57.80-119.1)	*<0.001*
		49.46 (25.04-121.7)		*0.005*

Values are given as medians (range).

### Cytokines in association to laboratory findings

At baseline there was no correlation between proteinuria and levels of any of the cytokines whereas at follow-up, a positive correlation was documented for IL-23 and proteinuria (r = 0.34, p < 0.05). Patients with persisting urine-albumin excretion > 0.5 grams/day at follow-up had higher IL-23 as compared to patients with <0.5 grams/day (median 5.27 vs. 2.29 pg/ml, p = 0.05). The group of patients with very low-grade proteinuria at follow-up (urine albumin <0.2 g/day), had more significantly lower levels of IL-23 (p = 0.01) compared to patients with ≥ 0.2 g/day.

A weak inverse correlation was documented at baseline for C3 and IL-10 (r = −0.31, p < 0.05) and at follow up for C3 and IL-23 (r = −0.44, p < 0.05), TNF-α (r = −0.36, p < 0.05) and IFN-γ (r = −0.30, p < 0.05) whereas no correlations were found for C4.

There was no correlation between serum creatinine and any of the investigated cytokines.

### Cytokines in association to histopathology

First we studied the cytokines in association to histopathological findings at both first and repeated biopsies. There was no difference in baseline cytokine levels between patients with class III, IV (proliferative nephritis, PN) vs. class V (membranous nephritis, MN) at baseline biopsies.

**Figure 1:**
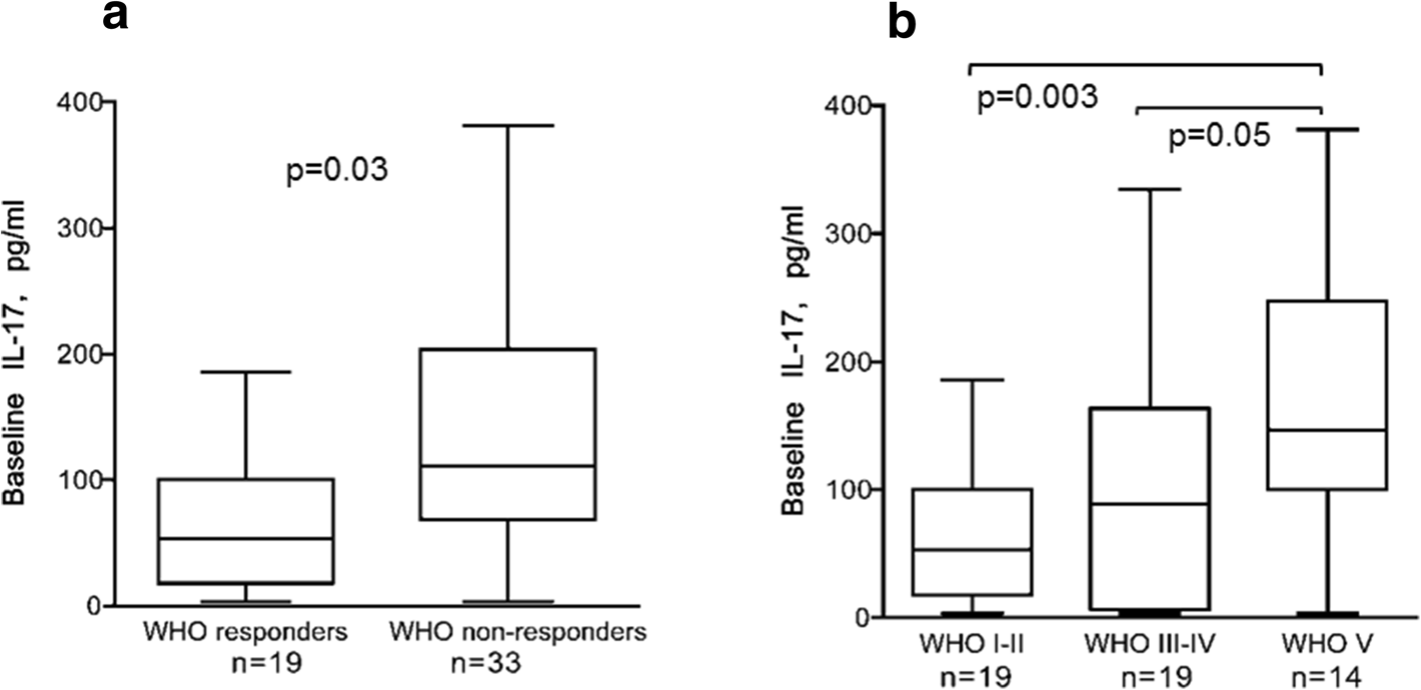
**Baseline levels of IL-17 were higher in patients with an unfavourable histopathological outcome. (a)** Baseline levels were significantly higher in patients who had a persisting active nephritis (WHO class III, IV or V) at follow-up, i.e. WHO non-responders, vs.WHO responders (WHO I or II). **(b)** Baseline levels of IL-17 in patients in relation to histopathology at follow-up. The patient with vasculitis at repeat biopsy was here included in the class III-IV group. The highest levels were seen in patients with a memranous nephritis, WHO class V, at follow-up. Boxes limits show 25^th^ to 75^th^ percentile and median values are marked inside the boxes.

Patients with a poor histopathological response, i.e. with a persisting active nephritis after immunosuppressive treatment, had significantly higher baseline levels of IL-17 compared to patients with WHO I or II at follow-up (median 111.0 vs. median 53.1 pg/ml) (p = 0.03) (Figure 1a). The highest baseline levels of IL-17 were seen in patients with MN at repeat biopsy (median 146.5 pg/ml) (Figure 1b). No significant differences in baseline levels of any of the other cytokines were found between histopathological responders and non-responders.

**Figure 2:**
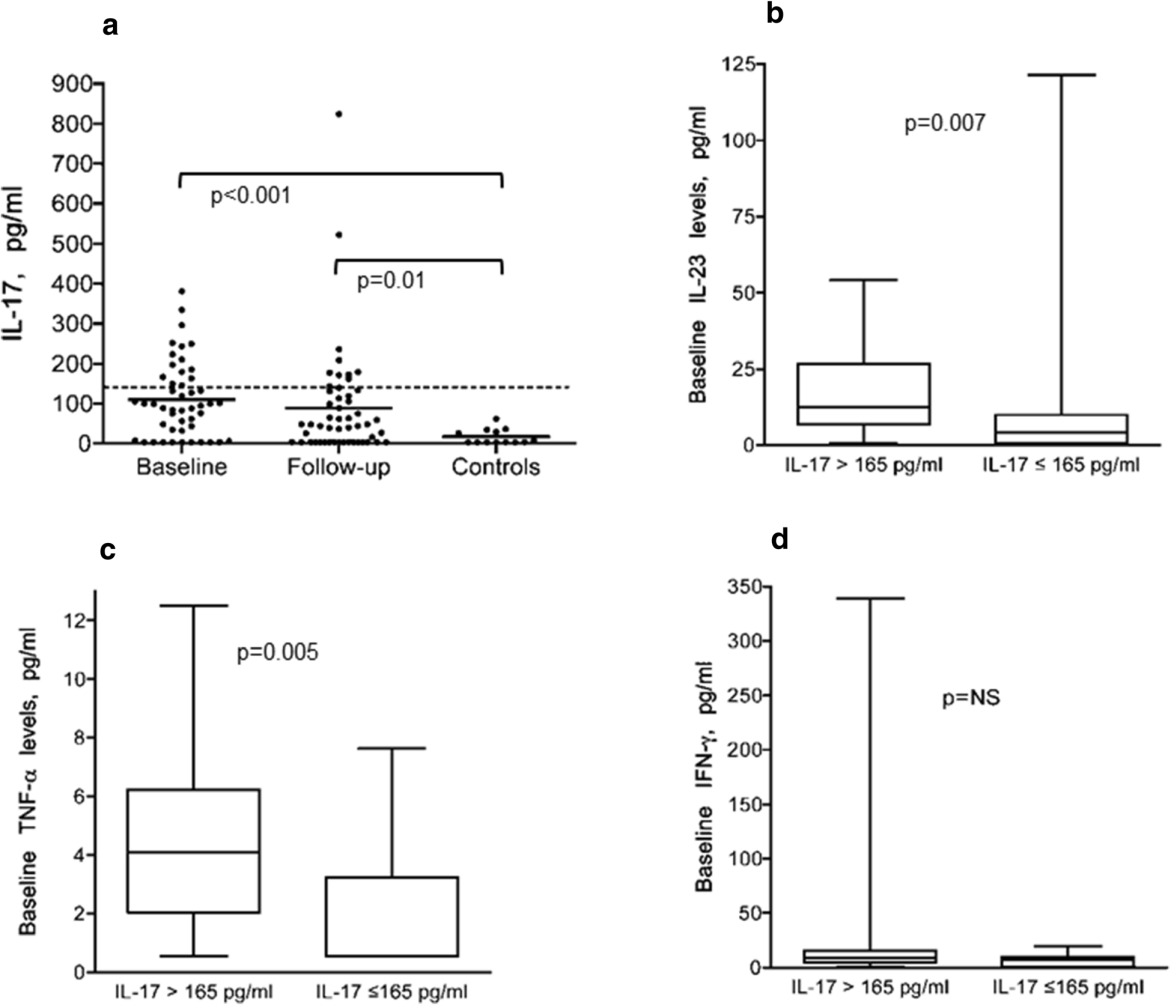
**Serum levels of IL-17 in patients and controls. Patients with the highest baseline levels had higher levels of IL-23, TNF-α and IFN-γ. (a)** Serum levels of IL-17 in patients at baseline and follow-up in patients and levels in controls (medians 97.4, 48.0 and 3.3 pg/ml respectively). The dotted line denotes the upper 25% of IL-17 levels (above 165 pg/ml). The patients with the highest baseline levels of IL-17 (>165) pg/ml had higher levels of IL-23 as shown in **(b)**, TNF-α **(c)** and IFN-γ **(d)** vs. those with IL-17 levels < 165. Boxes limits show 25^th^ to 75^th^ percentile and median values are marked inside the boxes.

**Figure 3:**
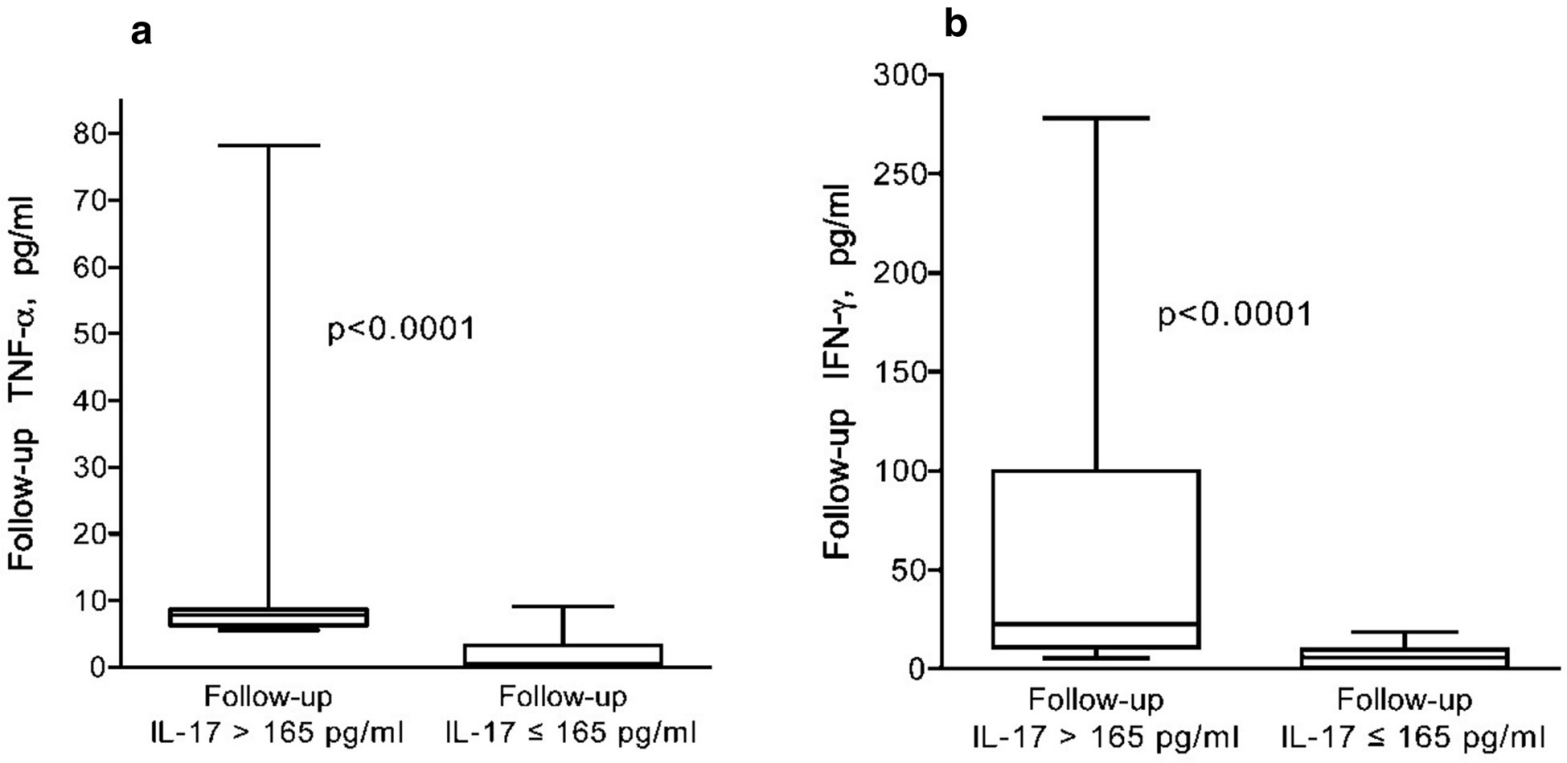
**Patients with persisting high levels of IL-17 at follow-up had higher levels of TNF-α and IFN-γ.** Patients with persisting high levels of IL-17 (>165 pg/ml) had significantly higer levels of TNF-α **(a)** and IFN-γ **(b)** vs those with IL-17 < 165 pg/ml. Boxes limits show 25^th^ to 75^th^ percentile and median values are marked inside the boxes.

Patients displayed higher IL-17 both at baseline (p < 0.001) and follow-up (p = 0.01) compared to controls (Figure 2a). Patients with the highest levels of IL-17 at baseline (arbitrarily defined as >165 pg/ml and encompassing the upper quartile), also had higher levels of IL-23 (p = 0.007), TNF-α (p = 0.005) and IFN-γ (ns) vs. those with IL-17 levels <165 pg/ml (Figure 2b, c and d). Of these patients, 11/13 (85%) were histopathological non-responders. At follow-up, 8/52 patients had persistently high IL-17 levels as defined above (Figure 2a), this subgroup also had higher levels of TNF-α (p = 0.0001) and IFN-γ (p = 0.0006) (Figure 3a and b) and were all histopathological non-responders (WHO III n = 4, WHO V n = 3 and one with glomerular vasculitis).

### Cytokines in association to clinical response

We also analysed cytokine levels in association to clinical response according to the definition used.

**Figure 4:**
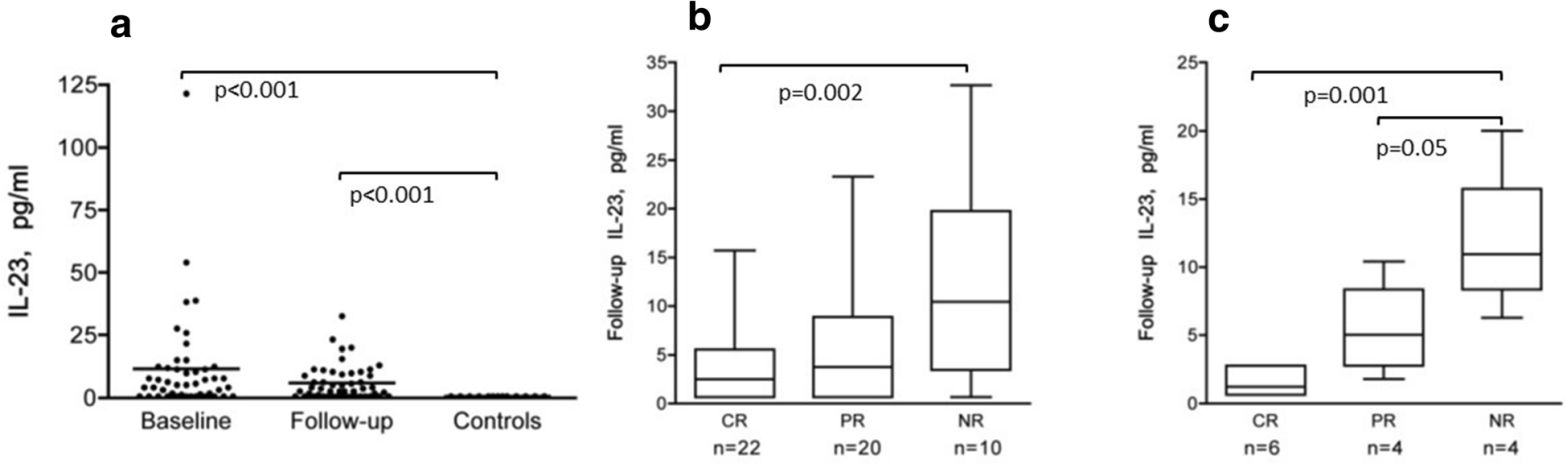
**Follow-up levels of IL-23 were higher in patients with an unfavourable BILAG response. (a)** Serum levels of IL-23 in patients at baseline and follow-up in patients and levels in controls (medians 5.5, 3.3 and 0.7 pg/ml respectively). **(b)** Levels of of IL-23 in all patients were significantly higher in BILAG non-responder (NR) patients vs. partial- (PR) and complete responders (CR), this was most pronounced in the group of patients with WHO class V at follow-up as shown in **(c)**. Boxes limits show 25^th^ to 75^th^ percentile and median values are marked inside the boxes.

Patients displayed higher IL-23 levels compared to controls both at baseline (p < 0.001) and follow-up (p < 0.001) (Figure 4a). At follow-up, BILAG-non-responders (NR) had significantly higher levels of IL-23 (median 10.6 pg/ml) vs. complete responders (CR) (median 2–6 pg/ml, p = 0.02) (Figure 4b). This was even more pronounced in the subgroup of patients with MN at follow-up (n = 14), in which the non-responding patients had higher IL-23 (median 10.9 pg/ml) vs. both PR (median 5.0 pg/ml, p = 0.05) and CR (median 1.2 pg/ml, p = 0.01) (Figure 4c).

No significant difference between CR-, PR- and NR-patients was found for any of the other cytokines.

### Correlations between cytokines

At baseline, IL-17 correlated with TNF-α (r = 0.63, p < 0.05), IFN-γ (r = 0.49, p < 0.05) and IL-23 (r = 0.30, p < 0.05). There was also a correlation between TNF-α and IFN-γ (r = 0.71, p < 0.05).

At follow up, IL-17 correlated with IFN-γ (r = 0.80, p < 0.05) and TNF-α (r = 0.79, p < 0.05). There was also a correlation between TNF-α and IFN-γ (r = 0.80, p < 0.05).

### Immunohistochemistry

**Figure 5:**
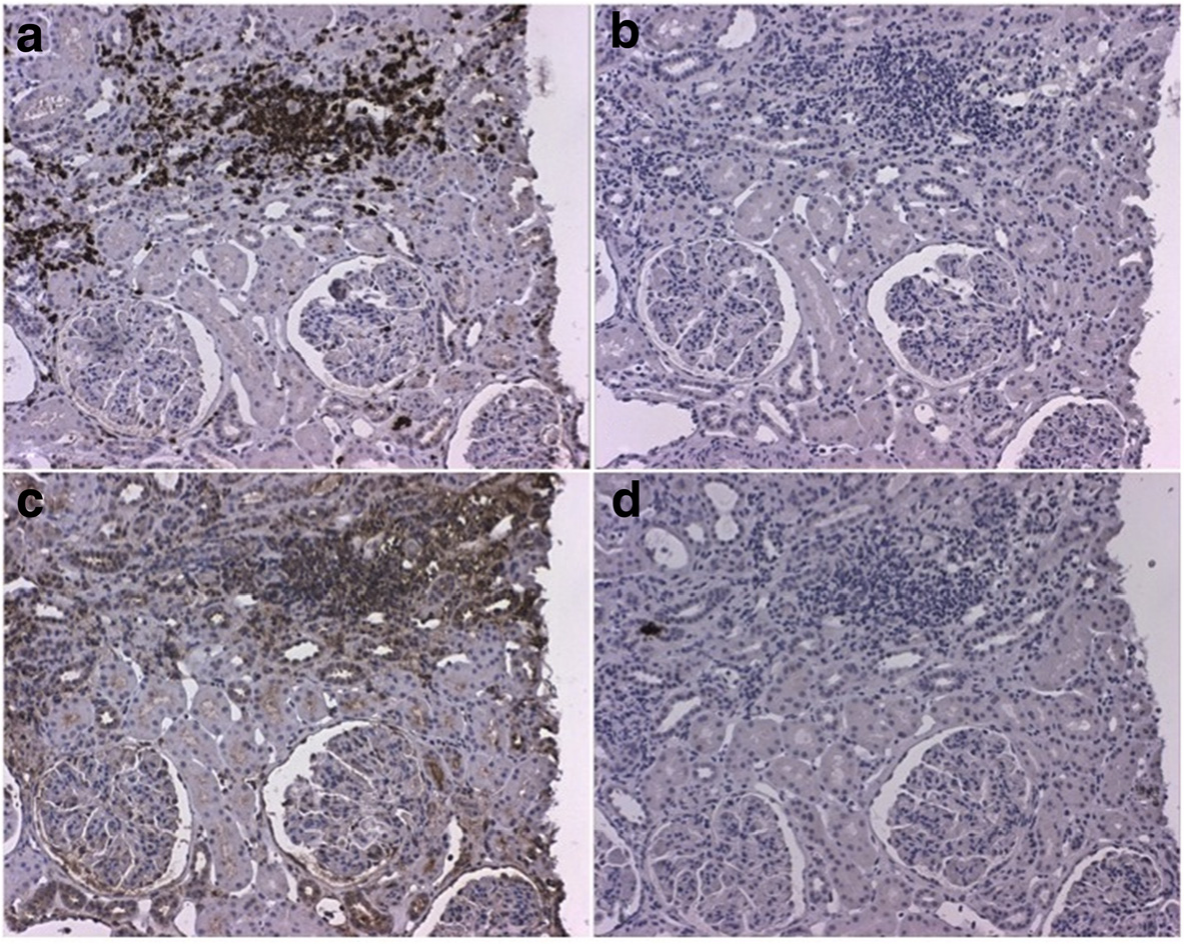
**Immunostaining of IL-17 in renal tissue.** The figure demonstrates a kidney biopsy from a patient with lupus nephritis WHO class V. Representative micrographs displaying **(A)** an inflammatory infiltrate with T cells as demonstrated by a positive CD3-staining and in **(B)** the same infiltrate from a consecutive section stained with irrelevant isotype control antibody. In **(C)**, IL-17 staining is demonstrated, predominantly found in the inflammatory infiltrate shown in A, and **(D)** demonstrates staining with the corresponding isotype control antibody. Original magnifications: 12.5×.

Immunostaining demonstrated expression of IL-17 in all the 6 examined biopsies from LN patients (4 with class V and 2 with class IV). The staining was most pronounced in areas of inflammatory infiltrates of CD3+ T-cells (Figure 5). There was no clear difference observed in the amount of IL-17 comparing PN and MN. In renal tissue from control kidney biopsies, IL-17 staining was negative (data not shown).

## Discussion

High baseline levels of IL-17 were here demonstrated as a marker of inadequate histopathological outcome after immunosuppressive therapy in LN, indicating that high IL-17 production could be associated with a severe, or therapy resistant, disease phenotype. Furthermore, high levels of IL-23 were associated with a poor clinical outcome. Our findings thus extend previous studies of an important role for the IL-23/IL-17 axis in LN, here also demonstrated to associate with therapeutic outcome. Our study also confirms and strengthens previous reports on increased levels of IL-6, IL-10, TNF-α, IFN-γ, IL-17 and IL-23 as well as low TGF-β in lupus patients [6,25-27].

There is increasing evidence for IL-17 involvement in the pathogenesis of SLE. Previous studies have shown increased number of IL-17 producing cells in peripheral blood [13], including both CD4+ Th17-cells and DN T-cells [16], and increased serum levels of IL-17 in SLE patients [12]. In LN, DN T-cells have been proposed to be the major source for IL-17, supported by studies demonstrating that DN T-cells infiltrate the kidneys of LN patients and produce high amounts of IL-17 and IFN-γ [16]. Similar findings have also been shown in experimental studies, demonstrating DN T-cells in inflammatory infiltrates and increased IL-17 and IFN-γ in kidneys in murine lupus models [28]. Our findings of high levels of IL-17, in combination with elevated IFN-γ, in patients with an unfavourable histopathological outcome, provide a context to previous observations.

Immunostaining revealed IL-17 expression in renal tissue from LN patients, most pronounced in areas with infiltrating T-cells, this confirms previous findings and clearly indicates that IL-17 is involved in the inflammatory process in the renal tissue.

High baseline IL-17 was associated with a poor histopathological response, with significantly higher levels among non-responder patients. Interestingly, the subgroup of patients with high baseline levels in combination with persistently high or increasing levels, all had an active nephritis at follow-up. Although this group only consisted of eight patients, this indicates that patients with high IL-17 may represent a group of severe LN with a strong T-cell component in whom current treatments may be insufficient or inadequate. This notification is further emphasized by the observation that the patients with the highest baseline IL-17 also had higher TNF-α, IFN-γ and IL-23, and of whom a majority was non-responsive to treatment.

High IL-23 levels were shown to be associated with poor clinical response as estimated by BILAG. Interestingly, patients with MN at follow-up had the highest levels of IL-17. In addition, among clinical NR-patients, the highest levels of IL-23 were seen in MN. Taken together, the findings may indicate a particular role for IL-23/IL-17 in MN but warrant further studies in larger patient populations.

As biomarkers currently available for assessing renal activity or response to therapy do not always reflect the actual inflammatory activity in renal tissue [29-31] there is a clear need for novel tools to assess activity in LN. Although new biomarkers both in blood and urine, including several cytokines, have been studied in LN, none have yet replaced conventional clinical parameters to monitor renal disease activity in clinical practise. It has also been proposed that a combination of biomarkers should be used to provide the best specificity and sensitivity [32-34]. Our findings of high IL-17 and IL-23 in relation to treatment outcome in LN suggest that these cytokines could be evaluated as novel biomarkers for severe disease.

Evaluation of response in LN is difficult, and despite extensive efforts, there are currently no generally accepted response criteria available. Most studies in LN use proteinuria as the most important outcome for response, here we used response according to change in renal BILAG which include change in proteinuria. Recently a consensus statement on the terminology used in LN was published, also proposing that the term remission should only be used based upon findings on a renal biopsy [35]. Having repeated biopsies available, we evaluated both clinical and histopathological response, thus giving unique opportunities to define response on a tissue level.

Introduction of new treatment strategies and improved global care have improved the prognosis in LN. However, a proportion of patients do not respond satisfactory to standard immunosuppressive treatment. Side effects of currently used therapies are a concern and long-term outcome remains unsatisfactory [36,37]. Accordingly, there is definitely a need for improved and more targeted treatment strategies in LN. Increased knowledge about the pathogenesis for SLE, in combination with the successful use of biologics in other autoimmune diseases, have paved the way for the development of biologic agents targeting different cytokine pathways also in SLE and thus, many anti-cytokine therapies for SLE are currently studied [38]. Trials targeting the IL-23/IL-17 pathway are underway for various autoimmune diseases, and targeting IL-23 (p40) is already in use for psoriasis, but the efficacy of these therapies in lupus remains to be studied [5]. Interestingly, a recent report documented beneficial effects of treatment of subacute cutaneous lupus with ustekinumab, a human monoclonal antibody that binds to the p40 subunit of IL-23 and inhibits its biological effects [39], indicating that blocking of IL-23 may be useful in lupus patients.

The main focus of the study has been lupus nephritis where non-renal manifestations have not been taken into consideration but may, at least to some extent, have influenced the cytokine levels. However, the increased IL-17 staining in the renal biopsies clearly points to its involvement in LN. Differences in treatment regimens may also have impact on study results. In this study most patients received high-dose CYC, however nowadays the low-dose Euro-lupus regimen [40] is standard of care for LN. There were no differences in cytokine levels comparing MMF and CYC, but whether different dose regimens of CYC may have impact on cytokine levels are not known.

## Conclusions

This study clearly supports a role for IL-23/IL-17 axis in LN and indicates that this cytokine pathway can be useful as biomarkers for renal disease activity and for predicting response to immunosuppressive treatment, especially in patients with a membranous phenotype. If monitoring of IL-17/ IL-23 could be helpful in predicting renal flares or could be used as predictors of long-term prognosis needs to be further studied. The findings may also point to possible targets for new treatment strategies in LN.
